# Injections of Pilocarpine in Edema of the Glottis

**Published:** 1882-03

**Authors:** 


### INJECTIONS OF PILOCARPINE IN EDEMA OF THE GLOTTIS.

At a recent meeting of the Societe de Therapeutique (*Bull. et Mem. de la Soc. de Therap.*) Dr. Paul read a paper by Dr. F. Sorel, giving an account of a case of edema of the glottis where other remedies having failed, one centigram (one sixth of a grain) of pilocarpine dissolved in a cubic centimeter of water was given. Slight perspiration resulted, with profuse salivation with the expulsion of large plugs of thick muco-pus. The relief was immediate. The injection was repeated a few hours later, and the next day two centigrams (one third of a grain) of the nitrate of pilocarpine were injected, giving rise to salivation and sweating.—*Med. Times.*

